# Generalized anxiety disorder in primary care: mental health services use and treatment adequacy

**DOI:** 10.1186/s12875-015-0358-y

**Published:** 2015-10-22

**Authors:** Pasquale Roberge, François Normand-Lauzière, Isabelle Raymond, Mireille Luc, Marie-Michèle Tanguay-Bernard, Arnaud Duhoux, Christian Bocti, Louise Fournier

**Affiliations:** Department of Family Medicine and Emergency Medicine, Faculty of Medicine and Health Sciences, Université de Sherbrooke, 3001,12th Avenue North, Sherbrooke, QC J1H 5 N4 Canada; Division of Neurology, Faculty of Medicine and Health Sciences, Université de Sherbrooke, 3001,12th Avenue North, Sherbrooke, QC J1H 5 N4 Canada; Faculty of Nursing, Université de Montréal, Pavillon Marguerite-d’Youville, C.P. 6128 succ. Centre-ville, Montreal, QC H3C 3 J7 Canada; CRCHUM (Centre de recherche du Centre Hospitalier de l’Université de Montréal), Université de Montréal, Pavillon Édouard-Asselin, 264, boul. René-Lévesque Est, Montréal, QC H2X 1P1 Canada

**Keywords:** Generalized Anxiety Disorder, Quality indicators, Treatment Adequacy, Primary Care, Service Utilization, Psychotherapy, Pharmacotherapy

## Abstract

**Purpose:**

Generalized Anxiety Disorder (GAD) is a common mental disorder in the primary care setting, marked by persistent anxiety and worries. The aims of this study were to: 1) examine mental health services utilisation in a large sample of primary care patients; 2) explore detection of GAD and minimal standards for pharmacological and psychological treatment adequacy based on recommendation from clinical practice guidelines; 3) examine correlates of treatment adequacy, i.e. predisposing, enabling and needs factors according to the Behavioural Model of Health Care Use.

**Methods:**

A sample of 373 adults meeting DSM-IV criteria for Generalized Anxiety Disorder in the past 12 months took part in this study. Data were drawn from the “Dialogue” project, a large primary care study conducted in 67 primary care clinics in Quebec, Canada. Following a mental health screening in medical clinics (n = 14833), patients at risk of anxiety or depression completed the Composite International Diagnostic Interview-Simplified (CIDIS). Multilevel logistic regression models were developed to examine correlates of treatment adequacy for pharmacological and psychological treatments.

**Results:**

Results indicate that 52.5 % of participants were recognized as having GAD by a healthcare professional in the past 12 months, and 36.2 % of the sample received a pharmacological (24.4 %) and/or psychological treatment (19.2 %) meeting indicators based on clinical practice guidelines recommendations. The detection of GAD by a health professional and the presence of comorbid depression were associated with overall treatment adequacy.

**Conclusions:**

This study suggests that further efforts towards GAD detection could lead to an increase in the delivery of evidence-based treatments. Key targets for improvement in treatment adequacy include regular follow up of patients with a GAD medication and access to psychotherapy from the primary care setting.

## Background

Generalized Anxiety Disorder (GAD) is a common mental disorder marked by persistent anxiety and worries, which are excessive and difficult to control, as well as multiple psychological and physical symptoms [1]. GAD often has a chronic course [2–6] with a lifetime prevalence rate for DSM-IV criteria estimated at approximately 6 % [7–10]. Persons suffering from GAD present significant impairments in work, social and family functioning, and health-related quality of life [11–16]. There is also increasing evidence regarding the economic burden of GAD in terms of lost work productivity and medical costs due to high utilization of medical services [8, 15, 17, 18]. GAD is highly associated with comorbid psychiatric disorders, with major depressive disorder being the most frequent [2, 3, 11, 19–21], and comorbid physical illness [22].

**Fig. 1:**
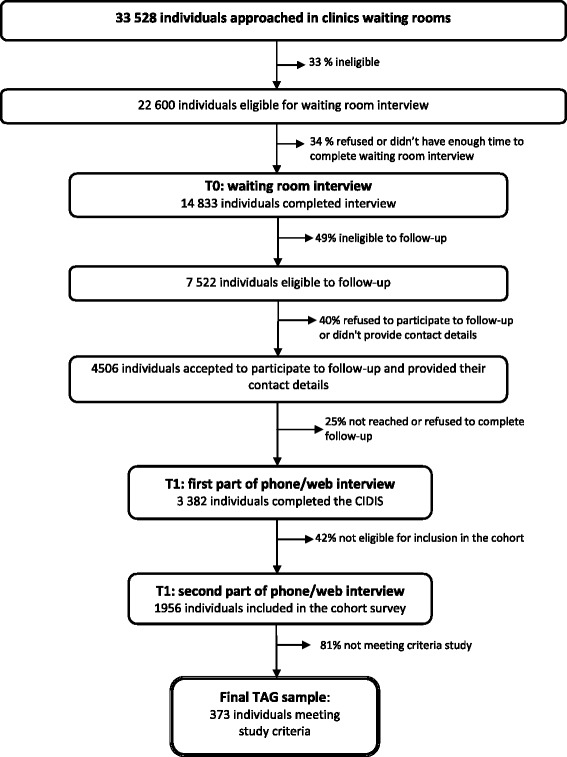
Recruitment flow-chart, Dialogue Project, 2008

Previous research has exposed the challenges to the detection and treatment of mental disorders in primary care [23]. Research has typically shown low rates of recognition of GAD by primary care providers [8–10, 24–28]. For GAD in particular, it has been suggested that under-recognition may be due to vague somatic symptoms, to patient’s attribution of symptoms to physical problems, to an ill-defined diagnosis [2] and to the variety of clinical presentations [5], which may depend on the symptom overlap with comorbid psychiatric disorders and somatic diseases [2, 29–32].

Patients with GAD often consult in the primary care setting [8] and it is generally agreed that most cases should be treated in primary care [2]. Clinical practice guidelines recommend either pharmacological treatments (e.g. SSRIs and venlafaxine) or cognitive behaviour therapy as first choice treatments for GAD, and long-term therapy may be needed to prevent relapse [33–35]. Previous studies have reported treatment adequacy rates for patients with GAD ranging from 24.6 % to 42.5 % in epidemiological surveys in Canada, United States, Spain and Australia [17, 36–38], 44.2 %-43.8 % [39, 40] in clinical studies in the United States and 49.5 % in a primary care sample in the Netherlands [41]. While GAD shares common characteristics with other anxiety disorder, we cannot assume that the determinants of potentially adequate psychological and pharmacological treatments are similar across anxiety disorders. For instance, research has shown that perceived need for treatment, help-seeking behaviour, service utilization, as well as recognition and treatment of common mental disorders by health care professionals vary across common mental disorders, which may impact on the probability of receiving potentially adequate treatments [38].

Despite the burden of GAD, data on patterns of service utilization, detection rates and treatment gap are lacking for this common anxiety disorder in the primary care setting. While common mental disorders treatment adequacy data is useful for policy planning, further data on disorder-specific treatment adequacy in primary care is also needed to inform quality improvement initiatives and reduce the gap between guidelines’ recommendations and clinical practice for GAD. We sought to examine mental health services utilisation in a large sample of primary care patients of Québec, Canada. A second aim of our investigation was to explore detection of GAD and minimal standards of pharmacological and psychological treatment adequacy for GAD based on recommendation from clinical practice guidelines. To our knowledge, only one study has provided estimates of treatment adequacy for GAD with a large naturalistic primary care sample in Europe [41]. The primary care perspective, in contrast with epidemiological survey or clinical trials, is important because these patients are in contact with the health care system and anxiety disorders are generally treated in the primary care setting [38, 42, 43]. As in previous studies, we expected low rates of recognition and evidence-based treatment of GAD. Finally, we also sought to examine individual-level correlates of treatment adequacy using Andersen’s Behavioural Model of Health Care [44] to examine the contribution of conceptually distinct predisposing factors, enabling factors and need for care factors associated with treatment adequacy for GAD. The widely used framework considers individual and contextual characteristics associated with service use. We expected that both GAD recognition and the presence of comorbid depression would improve the likelihood of treatment adequacy for primary care patients. Major depression is associated with increased severity and functional impairment of GAD [45].

## Methods

### Study setting, participants and data collection

Data were drawn from the “Dialogue” project, which included a large cohort study to examine mental health status, service utilization and experience of care of primary care patients with anxiety or depressive disorders [46]. Data for the current study were drawn from the waiting room screening questionnaire (T0) and the first telephone/web interview (T1). The study received the approval of all regional research ethics committees (Agence de santé de des services sociaux de Montréal; Centres de santé de des services sociaux de Chicoutimi, Sherbrooke, Gatineau, Laval, Saint Jérome, Jeanne-Mance, Lac-Saint-Jean-Est, Pointe-De-L’ile, Bordeaux-Cartierville-Saint-Laurent, Therese-De-Blainville, Pierre Boucher, Haut-Richelieu-Rouville, Baie des Chaleurs, La Pommeraie; Hospital Notre-Dame and Hospital Sacré-Coeur). Study participants provided written informed consent.

### Waiting room interview (T0)

Participants were recruited in 2008 in the waiting rooms of 67 primary care medical clinics (T0) during randomly chosen periods. Patients were invited by a lay-interviewer to complete a brief self-administered screening questionnaire if they met the following inclusion criteria: 1) age 18 years or older; 2) consulting a general practitioner for themselves; 3) able to complete a questionnaire in French or English. From the 22 600 eligible patients approached, 67.4 % (n = 14 833) completed the questionnaire. The screening questionnaire included general questions about socio-demographic characteristics, overall health status, consultations with health care providers, psychotropic medication, as well as the Hospital Anxiety and Depression scale (HADS) [47, 48].

### Telephone/web structured interview (T1)

Patients were invited to participate in the first part of T1 (n = 7 522) structured interview if their usual care source was one of the participating clinics and if they met at least one of the following inclusion criteria: i) elevated anxiety and/or depressive symptoms; ii) anxiety and/or depression medication in the past 12 months; iii) depressive and/or anxiety disorders diagnosis made by a physician; iv) consultation for mental health problems in the past 12 months. Among them, 4 506 (59.9 %) accepted to participate to the follow-up and provided their contact details. After 2–4 weeks, we were able to contact by telephone and/or email 3 382 (75.1 %) individuals and they completed either the telephone (70.8 %) or web (29.2 %) questionnaires. The first part of the questionnaire comprised a brief, structured psychiatric interview for lay interviewers that indicated the extent to which symptoms met the DSM-IV diagnostic criteria for common mental disorders, i.e. the Composite International Diagnostic Interview–Simplified (CIDIS) [49].

The interview then continued (second part of T1) with the 1 956 people meeting any of the following criteria: i) meeting DSM-IV criteria for generalized anxiety disorder, panic disorder, agoraphobia, social phobia or depression in the last 12 months; ii) a high level of anxiety or depression symptoms combined with medication use, diagnosis by a health care professional, or DSM-IV criteria for anxiety or depression in the past 24 months. This second part of the interview included questions on experience of care, services utilization for emotional reasons, medication use for anxiety or depressive symptoms, perceived needs for care and socio-demographic data. For the present study, the final sample included 373 adults meeting the criteria for GAD during the 12 months preceding the survey Fig. 1.

### Indicators for the detection of GAD, service utilization and treatment adequacy

Indicators for the detection of GAD in our sample were defined as: 1) reporting a diagnosis of GAD by a physician over the lifetime; 2) being informed in the past 12 months by a healthcare professional that they were suffering from GAD. Indicators for service utilization comprised: 1) being hospitalized for at least one night for mental health reasons in the past 12 months; 2) consulting at least one health professional for mental health reasons in the past 12 months, including a general practitioner, a psychologist, a social worker, counsellor or psychotherapist, a psychiatrist, a nurse or a medical specialist (other than a psychiatrist). Quality indicators for pharmacological and psychological management of GAD were established from the *Clinical practice guidelines*: *Management of anxiety disorders* published in 2006 by the *Canadian Psychiatric Association* [34]. We also built on previous research on treatment adequacy for anxiety and depression to expand our treatment adequacy indicators and explore other aspects of care that were not explicitly advocated in the guidelines [39, 40, 46, 50–52]. Two principal definitions of potentially adequate treatment were developed for the management of GAD. First, potentially adequate pharmacological treatments were defined as: receiving a first-line, second-line or third-line agent for GAD at an adequate dosage, plus at least 3 consultations with a general practitioner or psychiatrist. Benzodiazepines were not included as adequate medication because they are only recommended as a short-term adjunct medication for most patients. Second, the criteria for adequate psychotherapy were defined as: at least 12 psychotherapy sessions with the same mental health professional and a cognitive behavioural treatment. Overall adequacy was then defined as meeting criteria for either one or both psychological or pharmacological treatment adequacy. The comprehensive list of quality indicators regarding service use, GAD detection and treatment is presented in Table 2.

### Individual-level patient characteristics

Patient characteristics were conceptually grouped into predisposing, enabling, and needs factors according to Andersen’s Behavioural Model of Health Services Use [44]. Predisposing factors included sex, age (18–24; 25–44; 45–59; 60+), educational attainment (high school or less; college degree; university degree) and marital status (married/living together; separated/divorced/widowed; single). Enabling factors included perception of economic situation (financially secure; sufficient; poor/very poor), having a family physician and having private or collective insurance coverage for medication or complementary health services. Need for care factors included suffering from a depressive disorder or other co-morbid anxiety disorders in the past 12 months and the number of chronic physical illnesses (0, 1, 2, 3 or more).

### Statistical analysis

Descriptive analyses were conducted to examine socio-demographic and clinical characteristics of the sample, as well as overall service use and quality indicators for primary care patients with GAD. Analyses were carried out for the following adequacy indicators: 1) pharmacological treatment, 2) psychological treatment, 3) pharmacological and/or psychological treatment. We first calculated bivariate associations between aforementioned variables and treatment adequacy using logistic regressions. Given the hierarchical structure i.e. patients within primary care clinics, 2-level multilevel analyses were conducted using *glmer*. The empty model was first assessed with only clinical ID’s to identify the degree of association among observations within the clusters. In a second step, multilevel models were assessed for each adequacy with clinical ID’s as a random intercept [53]. Variables integrated in the models were based on the level of significance in the bivariate models (p < = .10). Although not reaching this criterion, sex was included as a control variable. Statistical analyses were performed using SPSS 20 statistical software and R 3.1.1.

## Results

### Patient characteristics

Table 1 summarizes the individual characteristics of the 373 participants meeting DSM-IV criteria for GAD in the 12 months preceding their interview. Participants with GAD were predominantly female (76.7 %). The mean age of participants was 42.2 years old (SD = 13.2) and more than 80 % were aged between 25 and 59 years. Slightly more than half were married or living with a partner (53.1 %) and more than half had completed a college or university degree. The sample was predominantly urban, approximately half was working or studying full-time (47 %) and over two thirds perceived their income as sufficient or felt financially secure.

**Table 1:** Individual Characteristics of Participants (*n* = 373)

CHARACTERISTICS	N	%
Sex (female)	286	76.7
Age group, years		
18-24	37	9.9
25-44	167	44.9
45-59	139	37.4
60 and over	29	7.8
Marital status		
Married/Living together	198	53.1
Separated/Divorced/Widowed	64	17.2
Single	111	29.8
Education level		
High school degree or less	173	46.4
Collegial degree	105	28.2
University degree	95	25.5
Patient perception of his income		
Financially secure	49	13.2
Sufficient	203	54.6
Poor/Very poor	120	32.3
Private medication insurance coverage (Yes)	245	65.9
Supplementary insurance coverage for complementary health services (Yes)	202	55.0
Has a family physician (Yes)	308	83.5
Comorbid anxiety disorder (social anxiety disorder, panic disorder, agoraphobia)	226	60.6
Social anxiety disorder	124	33.2
Agoraphobia	129	34.6
Panic disorder	131	35.1
Comorbid major depression	265	71.0
Comorbid chronic illnesses		
0	65	17.4
1	89	23.9
2	80	21.4
3 or more	139	37.3

The majority reported having a family physician, nearly two thirds had private medication coverage and over one half had access to supplementary insurance coverage for complementary health services. The mean age of onset of GAD was in the late twenties (28.9 years, SD = 13.2), and nearly two thirds had had GAD for over 5 years. The presence of psychiatric comorbidity was frequent: in the past 12 months, 71 % of participants also met the criteria for major depression and 60.6 % had a comorbid anxiety disorder. The majority of participants (82.6 %) experienced at least one chronic physical condition.

### Indicators of care

Table 2 presents descriptive data on our indicators of service utilization, detection of GAD as well as pharmacological and psychological treatments. The prevalence of service use for mental health problems in the past 12 months was high (89.5 %). The health professionals most frequently consulted were general practitioners (87.4 %), psychologists (53.8 %) and psychiatrists (28.5 %). The majority of participants (54.4 %) received a referral by a primary care physician to consult a mental health specialist, either a psychologist (40.8 %) or a psychiatrist (22.8 %). Over 1 in 10 participants were hospitalized at least one night for mental health reasons in the past 12 months.

**Table 2:** Service Use and Indicators of Services Received in the Past Twelve Months

INDICATORS	N	%
*Service use*		
Was hospitalized for at least one night for mental health reasons	42	11.3
Consulted at least one health professional for mental health reasons	334	89.5
General practitioner	292	87.4
Psychologist	179	53.8
Social worker/counsellor/ psychotherapist	118	35.4
Psychiatrist	95	28.5
Nurse	80	24.1
Other medical specialist	40	12.1
Detection by a physician of an anxiety disorder during life course	240	67.2
Detection by a healthcare professional of GAD in the past 12 months	191	52.5
*Pharmacotherapy*		
Received any psychotropic medication	239	64.1
SSRIs	117	31.4
Other	103	27.6
Benzodiazepines	92	24.7
Antipsychotics	44	11.8
MAOIs	22	5.9
Anticonvulsants	3	0.8
TCA	0	0
Received an evidence-based GAD medication	203	54.4
Received an evidence-based GAD medication at an adequate dosage	182	48.8
Received an evidence-based GAD medication at an adequate dosage, plus at least 3 consultations with a general practitioner or psychiatrist	99	26.5
Received an evidence-based GAD medication at an adequate dosage for at least six months plus at least 3 consultations with a general practitioner or psychiatrist^a^	91	24.4
*Psychotherapy*		
Any form of psychotherapy or counselling	202	54,3
Any form of psychotherapy or counselling lasting ≥ 15 minutes	175	48.1
Problem solving therapy	143	84.1
CBT	137	80.1
Interpersonal psychotherapy	97	58.8
Psychotherapy with ≥ 12 sessions with a same healthcare professional	84	23.1
Psychotherapy, CBT approach and ≥ 12 sessions with a same healthcare professional^b^	70	19.2
*Pharmacotherapy and*/*or Psychotherapy*		
Received an evidence-based pharmacological treatment and/or psychotherapy	135	36.2

^a^Our indicator for potentially adequate pharmacotherapy

^b^Our indicator for potentially adequate psychotherapy

Based on our indicators for the detection of GAD, 67.2 % of participants reported being informed by a physician that they were suffering from an anxiety disorder during their life course and 52.5 % specifically of GAD in the past 12 months by a healthcare professional.

Overall, 36.2 % of the sample received a pharmacological and/or psychological treatment qualified as adequate according to our indicators of care. For pharmacological treatments, nearly two thirds of the participants had received a psychotropic medication and SSRIs were the most frequently prescribed class of medication (31.4 %). The indicator of pharmacological treatment adequacy was met for 24.4 % of the sample based on medication, dosage, duration and follow up (see Table 2). For psychotherapy, over one half of participants reported obtaining some form of psychotherapy or counselling, and slightly less than one half obtained at least 15 minutes or more of psychotherapy or counselling. The most frequently reported psychotherapeutic approaches were problem-solving therapy and cognitive behavioural therapy (CBT). The indicator of psychological treatment adequacy was met for 19.2 % of the sample based on the number of sessions of psychotherapy by a same healthcare professional and exposure to a CBT approach.

### Factors associated with treatment adequacy

Table 3 presents the results of the multilevel logistic regression analyses showing predisposing, enabling and needs factors associated with adequate pharmacological and psychological treatments for the sample.

**Table 3:** Predisposing, Enabling and Needs Factors Associated with Treatment Adequacy

	Pharmacotherapy^a^			Psychotherapy^b^			Overall^c^		
	(*N* = 357)			(*N* = 345)			(*N* = 307)		
		Multivariate Associations (Final Model)			Multivariate Associations (Final Model)			Multivariate Associations (Final Model)	
	Bivariate Associations	OR (95 % CI)	*P*	Bivariate Associations	OR (95 % CI)	*P*	Bivariate Associations	OR (95 % CI)	*P*
	*P*			*P*			*P*		
Predisposing factors									
Gender (female)	.613	0.95 (0.52-1.76)	.873	.086	2.16 (1.00-4.68)	.052	.526	1.32 (0.77-2.31)	0.311
Age group									
25-44 (ref)					1.00				
18-24	.384			.475	0.90 (0.32-2.50)	.836	.581		
45-59	.181			.008	0.48 (0.25-0.92)	.026	.938		
60+	.806			.083	0.45 (0.12-1.72)	.246	.598		
Education									
High school degree or less (ref)		1.00			1.00				
Collegial degree	.790	0.86 (0.46-1.60)	.638	.705	0.75 (0.35-1.60)	.455	.723		
University degree	.091	0.56 (0.28-1.31)	.107	.003	1.96 (0.98-3.94)	.057	.337		
Marital status									
Single (ref)		1.00							
Married/Living together	.853	0.91 (0.48-1.72)	.775	.163			.568		
Separated/Divorced/Widowed	.001	2.99 (1.40-6.39)	.005	.114			.160		
Enabling factors									
Patient perception of income									
Poor/Very poor (ref)					1.00				
Sufficient	.810			.437	1.18 (0.57-2.48)	.651	.192		
Financially secure	.456			.018	2.03 (0.80-5.14)	.134	.813		
Private medication insurance coverage (Yes)	.302			.252			.483		
Private insurance coverage for complementary health services (Yes)	.612			.068	1.16 (0.61-2.21)	.658	.712		
Has a family physician (Yes)	.026	3.11 (1.30-7.43)	.011	.591			.069	1.90 (1.01-3.71)	0.051
GAD detected by health professional in the past 12 months (Yes)	<.001	3.89 (2.18-6.94)	<.001	.006	2.94 (1.56-5.52)	.001	<.001	3.14 (1.98-5.04)	<0.001
Needs factors									
Comorbid major depression	.028	1.68 (0.90-3.16)	.106	.201			.009	1.82 (1.09-3.08)	0.024
Comorbid anxiety disorder	.973			.022	0.51 (0.28-0.94)	.031	.135		
Comorbid chronic illnesses									
0	.154			.805			.161		
1	.121			.683			.104		
2	.359			.602			.743		
3 or more (ref)									
Random factor (Variance components)									
Clinic ID (Intercept)		0.11			0.00			0.00	

NOTES: Bivariate association’s threshold is *p* ≤ 0.10. If no ORs are written for a model, associate variables are not included in the corresponding model

Gender is included in all final models (all non-significant)

^a^Indicator defined as: adequate GAD medication at an adequate dosage, plus at least 3 consultations with a general practitioner or psychiatrist

^b^Indicator defined as: psychotherapy with a CBT approach and ≥ 12 sessions of at least 15 minutes with the same healthcare professional

^c^Indicator defined as: adequate pharmacological^a^ and/or adequate psychological^b^ treatment

### Pharmacological treatment adequacy

A total of 357 cases were analyzed for the pharmacological treatment adequacy status. Proportion of variance explained by the random effect (intra-class correlation (ICC)) of clinical IDs was 11 %. One predisposing factor, marital status, was associated the reception of adequate pharmacological treatment. Compared to single participants, those who were separated, divorced or widowed were three times more likely to obtain adequate pharmacological treatment (OR = 2.99; 95 % CI [1.40-6.39]). Participants who had a family physician were three times more likely to receive adequate pharmacotherapy (OR =3.11; 95 % CI [1.30-7.43]), and detection of GAD by a health professional also increased the odds of treatment adequacy (OR = 3.89; 95 % CI [2.18–6.94]).

### Psychological treatment adequacy

A total of 345 cases were analyzed for the psychotherapy treatment adequacy status. Table 3 presents the odd ratios and confidence intervals for the model’s significant predictors. Proportion of variance explained by the random effect (ICC) of clinical IDs was around 0 %. The predisposing factors associated with adequate psychological treatment included only age group. Belonging to the 45–59 age group, compared to 25–44 age group, was associated with decreased odds of receiving adequate psychological treatment (OR = 0.48; 95 % CI [0.25–0.92]). The only enabling factor associated with psychological treatment adequacy was the detection of GAD by a health professional (OR = 2.94; 95 % CI [1.56–5.52]). For needs factors, the presence of a comorbid anxiety disorder (OR = 0.51; 95 % CI [0.28–0.94]) reduced the odds of reception of an adequate psychological treatment.

### Overall adequacy

Three hundred seven cases were analyzed for the overall adequacy, meaning that an individual met criteria for either one or both psychological or pharmacological treatment adequacy. The ICC of clinical IDs was near 0 %. The detection of GAD by a health professional (OR = 3.14; 95 % CI [1.98–5.04]), an enabling factor, and the presence of comorbid depression (OR = 1.82; 95 % CI [1.09–3.08]), a needs factor, were associated with overall treatment adequacy.

We did not observe significant differences between clinics in the treatment adequacy models. With 373 individuals separated within 65 primary care medical clinics, the ICC can be as low as zero (only one individual sampled in a clinic) and with a mean of six patients/clinic there is not enough variance within each clinic for the multilevel models to be noticeably different. Furthermore, a likelihood ratio test statistic based on full and reduced models (data not shown) was used to compare the models with and without the random effect and the null hypothesis was not rejected. HADS scores and diagnosis were also integrated in the regression models (data not shown) but did not add any relevant effect.

## Discussion

Results of our study indicate that 89.5 % of GAD sufferers in our primary care sample had consulted at least one health care provider for mental health reasons in the past 12 months, typically a general practitioner or a psychologist. As the recruitment of primary care patients necessarily indicates that they are in contact with at least one clinician, but not necessarily for mental health reasons, the data provides an original perspective on quality of care for patients that are actually exposed to clinical practice, which offers the opportunity for clinicians to actually provide mental health care to their patients. The majority of patients had been recognized as having an anxiety disorder in their lifetime (67.2 %) and more specifically a GAD (52.5 %) by a health care professional in the past 12 months. While these recognition rates are higher than expected, they may be explained in our sample by high rates of mental health service use, long-term GAD symptoms and profiles of psychiatric comorbidity, particularly with major depression. This is consistent with previous research that showed that comorbid psychiatric disorders and symptom severity facilitate detection of anxiety disorders, while excessive worry may not be sufficient for health care professionals to recognize GAD as well as for patients to decide to seek mental health care [14, 26, 54–56].

In our sample, 36.2 % of patients with GAD reported patterns of service use that met overall minimal standards of treatment adequacy based on clinical practice guidelines recommendations in the past 12 months. While our results are within range of prior studies, more stringent criteria for treatment adequacy in our study could explain variations. As seen in previous studies for anxiety disorders [42], treatment adequacy rates were higher for pharmacotherapy (24.4 %) than for psychotherapy (12.9 %). While consultations with psychologists and psychotherapists were frequent, the number of treatment sessions with the same provider did not present sufficient treatment intensity to meet our treatment adequacy indicator. Pharmacological treatments were characterized by adequate medication and dosage in half of patients, but regular follow up was lacking. This could in part be explained by the long-term GAD symptoms for over two third of the sample, where close follow up may not be as compulsory when medication is stabilized. As seen in other studies [57], our data on medication use also suggested that a large proportion of patients were taking benzodiazepines in the long term, despite clear guidance that it should be a short-term adjunct medication [34].

We examined predisposing, enabling and needs factors associated to treatment adequacy for GAD. Detection of GAD by a health professional in the past 12 months was an enabling factor for both types of treatment adequacy, with patients being over three times more likely to receive adequate treatment, and suggests that improving recognition of GAD in primary care could lead to an increase in guideline-concordant care. Having a family physician was also an enabling factor for pharmacotherapy adequacy, which is most likely related to continuity of care and patient follow up. The presence of comorbid depression was also associated with overall treatment adequacy. Strong patterns of comorbidity between GAD and major depression have been observed in our study, as well as in clinical and community samples [14, 32], and major depression has been associated with treatment adequacy for anxiety disorders in a number of studies [41, 42]. However, the presence of a comorbid anxiety disorder reduced the odds of reception of an adequate psychological treatment.

A number of limitations should be considered in the interpretation of the findings. First, the current dataset is based on self-reported data from a cross-sectional primary care mental health survey and differences in the reporting of mental health service use in surveys compared to administrative data has been highlighted in previous studies [58, 59]. Second, our results offer a partial view of the correlates of treatment adequacy from service utilization data that could be complemented by research on perceived needs for care and provider and clinic characteristics [60]. Further analyses of Dialogue project data revealed that approximately 40 % of the participants perceived unmet needs for mental health care, in particular for psychotherapy [61]. Third, our indicators of treatment adequacy should not be interpreted as a straightforward criterion for evidence-based treatments, as patient preference, clinical expertise, help seeking behaviour and other patient, provider and system factors are associated with quality of care. We tried to get a sense of the provision of a full course of evidence-based psychotherapy by examining the number of sessions of psychotherapy or counselling with the same provider in the past 12 months comprising CBT components. A total of 12 sessions may arguably be too severe as low intensity interventions for anxiety disorders are gradually gaining empirical support [62]. Furthermore, we relied on self-reported type of psychotherapy and we did not assess specific cognitive behaviour therapy components, which according to research conducted by Stein *et al*. (2004, 2011), could have led to lower treatment adequacy rates [39, 40].

## Conclusion

Our study offers an original perspective on treatment adequacy for GAD with a large primary care sample, where participants are in contact with health care services and the assessment of mental disorders is independent of primary care provider diagnosis and treatment. Our findings suggest that GAD is often recognized in the context of real world primary care, and that over a third of patients are treated according to clinical guidelines recommendations. Detection of GAD is an important correlate of treatment adequacy, and this suggests that further efforts should be invested in specific GAD screening and diagnosis. Also, while it is advocated that GAD patients should be treated in primary care to reduce stigma and improve access to care, some of the main challenges to improving care will be to ensure that patients with a GAD medication obtain a regular follow up and also that patients have sufficient access to evidence-based psychotherapy. Having a family physician was associated with pharmacotherapy adequacy, and primary care providers could also be very influential in referring patients to psychotherapy, providing a lessening of the barriers in access to psychologists. The vast majority of patients with GAD seek care from general practitioners, and this is an optimal context for shared decision-making when a multiplicity of treatment choices and resources are available in the community for patients with anxiety disorders. These efforts could lead to improved detection and evidence-based treatment rates for GAD patients in the primary care setting.
